# On-package claims are imperfect signals of overall nutritional quality

**DOI:** 10.1038/s43016-026-01435-9

**Published:** 2026-09-24

**Authors:** Pei Zhou, Yuqing Zheng, Harry M. Kaiser, Miguel I. Gómez

**Affiliations:** 1 Charles H. Dyson School of Applied Economics and Management, Cornell University, Ithaca, NY USA; 2 Department of Agricultural Economics, University of Kentucky, Lexington, KY USA

**Keywords:** Economics, Social policy

## Abstract

On-package claims, including Minus, Plus, Natural and Functional claims, are widely used to communicate nutritional or health-related benefits. Using data on newly launched US food and beverage products from the Mintel Global New Products Database and the UK nutrient profiling model (NPM), we assessed how well these claims signal overall nutritional quality. While products with claims are often associated with more favourable nutrient profiles at the market level, this pattern is inconsistent across claim types and product categories. In particular, food products carrying trans fat claims and beverages carrying sodium claims often exhibit poorer overall nutritional quality than those without the claims. Component analyses suggest potential reformulation trade-offs and selective signalling. Moreover, even when products carrying claims have more favourable nutrient profile scores, many remain classified as ‘less healthy’ under the NPM. These findings indicate that attribute-based claims are imperfect signals of overall nutritional quality, with important implications for food labelling policy and consumer decision-making.

## Abstract (web summary)

Many food and beverage products contain on-package claims, such as ‘Low Calorie’, ‘High Protein’, ‘All Natural’ or ‘Heart Healthy’, to communicate nutritional or health-related benefits. This study finds that these claims do not consistently correspond to better overall product nutritional quality.

## Main

The relationship between food and health has attracted increasing attention in public health and policy. Diet is widely recognized as a key determinant of non-communicable diseases (NCDs) in global and national policy frameworks^1,2^. Evidence from epidemiological, clinical and population-level studies shows that dietary patterns and nutrient intake are strongly associated with health risks, including obesity, type 2 diabetes, cardiovascular disease, certain cancers and mortality^3–7^. However, dietary patterns in the United States remain suboptimal, with insufficient intake of beneficial nutrients such as dietary fibre and iron and excessive consumption of added sugars, refined grains, saturated fats and sodium^8^.

A range of policies have been introduced to encourage healthier eating and reduce the burden of NCDs. These strategies can be broadly classified into information-based measures and market-based interventions^9^. Among the former, packaging-based information tools, particularly on-package claims, are widely used to guide consumer choices. Improving the information environment in which consumers make food choices has therefore become an important policy focus.

The regulation of food labelling in the United States has evolved from preventing deceptive marketing to supporting informed dietary choices. Early legislation, including the Food, Drug, and Cosmetic Act (1938) and the Fair Packaging and Labeling Act (1966), established federal oversight of misbranding and standardized basic product information^10,11^. The Nutrition Labeling and Education Act (1990) marked a major shift by standardizing nutrient content and health claims and introducing the nutrition facts label^12^. Subsequent developments, including the Dietary Supplement Health and Education Act (1994) and judicial decisions such as Pearson versus Shalala (1999), expanded the scope of permissible claims while requiring clearer communication of scientific uncertainty^13,14^. More recent US Food and Drugs Administration (FDA) actions, including trans fat labelling requirements, the 2016 nutrition facts label revision and the 2024 update to the ‘Healthy’ claim, alongside proposed front-of-package labelling rules, reflect a continued shift towards nutrient profiling-based frameworks designed to guide consumers towards healthier choices^15–18^.

In parallel with public health initiatives, food and beverage manufacturers increasingly use on-package claims as influential marketing tools^19–21^ to highlight nutritional content, the presence or absence of specific nutrients, health benefits and production practices. These claims include both regulated categories (for example, nutrient content claims and structure/function claims under FDA oversight) and a broader set of claims that are not strictly defined within regulatory frameworks (for example, natural claims).

As a direct interface between products and consumers, on-package claims simplify complex nutritional information into salient cues that guide choices at the point of purchase^22,23^. Acting as heuristic signals, they shape perceptions and purchase decisions^24–30^. However, because many claims emphasize specific attributes rather than overall nutritional composition, they may not provide a complete representation of product healthfulness, giving rise to ‘health halo’ effects and systematic overestimation of nutritional quality^22,31,32^.

Previous studies have documented the widespread use of on-package claims and compared the nutritional quality of products with and without such claims^19,33–35^. However, these studies often relied on cross-sectional designs, providing only a static snapshot of the marketplace. They also tended to assess nutritional quality based on selected nutrients rather than composite nutrient profiling measures and focused on specific product categories or sectors. Moreover, natural and functional claims remain underexplored despite their growing prominence in the food and beverage market. As a result, it remains unclear whether on-package claims align with overall nutritional quality. Therefore, in this study, we addressed two research questions: (1) are products carrying on-package claims associated with more favourable overall nutritional profiles than products without such claims? (2) Do products carrying such claims meet nutrient profiling thresholds for overall nutritional quality?

To address this gap, we compared products with and without claims at both the market level and within product categories. As claims refer to specific attributes, whereas overall nutritional quality reflects a composite of multiple nutrients, they may provide only partial information about product healthfulness. We further examined heterogeneity across claim types, including underexplored natural and functional claims, and investigated potential mechanisms through component decomposition. We also employed a classification framework that distinguishes market-wide discrepancies, within-category discrepancies and aligned patterns, allowing us to assess when on-package claims may function as informative versus potentially misleading signals.

We assessed overall nutritional quality using the UK nutrient profiling model (NPM 2004–2005), which is widely used in food policy, marketing regulation^36^ and international nutrition research^37,38^. The model evaluates components to limit and components to encourage to derive a composite nutrient profile (NP) score, with higher scores indicating less favourable overall nutritional quality. Separate thresholds for foods and beverages are used to classify products as ‘less healthy’. We drew on data from the Mintel Global New Products Database (GNPD), covering newly launched packaged food and beverage products from 2015 to 2024 and providing detailed information on product characteristics, nutrition facts, ingredients and claims. Using GNPD’s consumer-facing claim classification, we focused on four major types of on-package claim: Minus (reduced levels of nutrients to limit), Plus (added or increased beneficial nutrients), Functional (emphasizing physiological or health benefits) and Natural (related to production characteristics or ingredient origins). In our regulatory mapping, GNPD’s Minus and Plus claims are mapped to the FDA category of nutrient content claims, Functional claims are mapped primarily to structure/function claims, while Natural claims are not strictly defined within US regulatory frameworks. Detailed definitions and mappings to US regulatory frameworks are provided in ‘Claims classification’ and Supplementary Table 1.

We found that on-package claims provide imperfect signals of overall nutritional quality. At the market level, products carrying claims generally had lower, more favourable NP scores than products without claims, but this pattern was not consistent across claim types or product categories. Moreover, even when products carrying claims had lower NP scores than products without claims, many still exceeded the NPM thresholds for classification as less healthy. Thus, attribute-based claims may provide incomplete or potentially misleading guidance when interpreted as indicators of overall product healthfulness.

## Results

The final dataset included 145,609 products, comprising beverages and 10 food categories of processed consumer packaged goods (see Methods and Supplementary Table 2 for category definitions). All analyses refer to this sample unless otherwise noted.

### Nutritional quality of food and beverages by category

Table 1 summarizes the nutritional quality of food and beverage products by category, as assessed using the NPM. It reports the number of new products, the average NP score, the share of products classified as less healthy under the NPM and component score breakdowns: A points reflect components the model aims to limit and C points reflect components the model aims to encourage.

**Table 1:** Nutritional quality of food and beverages by category, 2015–2024

Category	Number of new products	NP score^a^	Less healthy under NPM (%)^b^	A Points					C Points				
				Total A points	Energy	Saturated fat	Total sugar	Sodium	Total C points	Adjusted total C points^c^	Fruit, vegetables and nuts	Fibre	Protein
Bakery	15,559	14.06 (8.93)	80.76 (39.42)	16.99 (7.03)	4.37 (1.36)	4.78 (4.02)	4.17 (3.37)	3.67 (2.34)	5.27 (2.80)	2.94 (2.87)	0.00 (0.00)	2.00 (1.83)	3.26 (1.48)
Beverage	10,572	−0.34 (2.13)	32.36 (46.79)	0.92 (1.12)	0.03 (0.23)	0.03 (0.30)	0.83 (0.96)	0.04 (0.31)	1.26 (2.12)	1.26 (2.12)	0.95 (1.96)	0.07 (0.34)	0.24 (0.85)
Breakfast Cereals	5,037	7.66 (6.63)	73.97 (43.88)	13.19 (4.75)	4.31 (0.94)	1.52 (2.67)	4.33 (2.51)	3.04 (2.37)	8.55 (2.28)	5.52 (2.70)	0.00 (0.00)	4.36 (1.40)	4.19 (1.15)
Candy	13,219	18.39 (7.12)	93.24 (25.10)	20.04 (7.62)	4.89 (1.63)	6.02 (4.71)	8.40 (3.01)	0.73 (1.46)	3.92 (3.32)	1.65 (1.99)	0.00 (0.00)	1.63 (1.96)	2.29 (1.81)
Condiments	17,723	10.32 (6.75)	80.53 (39.60)	11.74 (6.17)	2.24 (2.34)	2.01 (3.16)	1.92 (3.05)	5.57 (3.61)	2.26 (3.08)	1.42 (2.18)	0.00 (0.00)	1.06 (1.71)	1.21 (1.80)
Dairy and Eggs	14,539	10.67 (10.37)	58.83 (49.22)	12.06 (9.00)	2.45 (2.20)	5.51 (4.48)	0.83 (1.66)	3.27 (3.60)	3.43 (2.11)	1.39 (1.97)	0.00 (0.00)	0.13 (0.58)	3.29 (2.02)
Desserts and Ice Cream	5,979	11.63 (6.92)	83.79 (36.85)	12.67 (6.37)	2.34 (1.37)	4.98 (3.86)	4.39 (2.38)	0.95 (1.92)	2.27 (2.06)	1.04 (1.81)	0.00 (0.00)	0.71 (1.28)	1.56 (1.34)
Meals and Side Dishes	21,218	3.96 (6.75)	40.14 (49.02)	7.83 (5.27)	2.21 (1.44)	1.77 (2.36)	0.23 (0.62)	3.61 (2.93)	5.16 (2.55)	3.86 (2.79)	0.00 (0.00)	1.63 (1.49)	3.53 (1.71)
Processed Fruit and Vegetables	3,260	−4.85 (3.42)	2.18 (14.60)	3.21 (2.87)	0.49 (0.90)	0.17 (0.75)	0.83 (1.59)	1.72 (1.86)	8.06 (2.92)	8.06 (2.92)	5.00 (0.00)	1.96 (1.77)	1.10 (1.36)
Processed Meat and Seafood	14,239	7.33 (8.92)	52.83 (49.92)	10.38 (6.87)	1.97 (1.33)	3.11 (3.50)	0.12 (0.50)	5.18 (3.56)	5.09 (1.06)	3.05 (2.61)	0.00 (0.00)	0.24 (0.83)	4.86 (0.76)
Snacks	24,264	12.65 (6.51)	90.38 (29.48)	16.34 (5.60)	5.08 (1.64)	4.10 (3.48)	2.83 (3.46)	4.34 (3.51)	7.23 (2.77)	3.69 (2.32)	0.00 (0.00)	3.40 (1.93)	3.83 (1.62)
All products	145,609	9.51 (9.12)	67.25 (46.93)	12.26 (8.06)	3.05 (2.25)	3.36 (3.89)	2.45 (3.42)	3.40 (3.38)	4.65 (3.26)	2.76 (2.80)	0.18 (0.93)	1.56 (1.94)	2.91 (2.04)

^a^The NP score is calculated by subtracting C points from A points. A higher NP score indicates that a product is less healthy. ^b^The percentage of products classified as less healthy under the NPM. The underlying indicator equals 1 if a product is classified as less healthy and 0 otherwise. The classification is based on product type: drinks with NP scores ≥1 and foods with NP scores ≥4 are classified as less healthy. ^c^If a product earns 11 or more A points, it cannot receive protein points unless it also earns at least 5 points for fruit, vegetables and nuts. Values in parentheses are standard deviations.

Categories such as Snacks, Meals and Side Dishes, Condiments and Bakery account for the largest number of product launches. Processed Fruit and Vegetables has the lowest average NP score (−4.85) with only 2.18% classified as less healthy, reflecting the lowest A points among all food categories, and the highest adjusted C points. Beverages follow (mean −0.34 with 32.36% classified as less healthy), reflecting relatively low levels of components to limit. In contrast, products in the Candy, Snacks, Desserts and Ice Cream, and Bakery categories show the highest NP scores (18.39, 12.65, 11.63 and 14.06), with 93.24%, 90.38%, 83.79% and 80.76% of products classified as less healthy, respectively. Meals and Side Dishes lies near the NPM threshold, reflecting lower total sugar and higher protein alongside higher sodium content.

### Trends in on-package claims and product nutritional quality

**Fig. 1:**
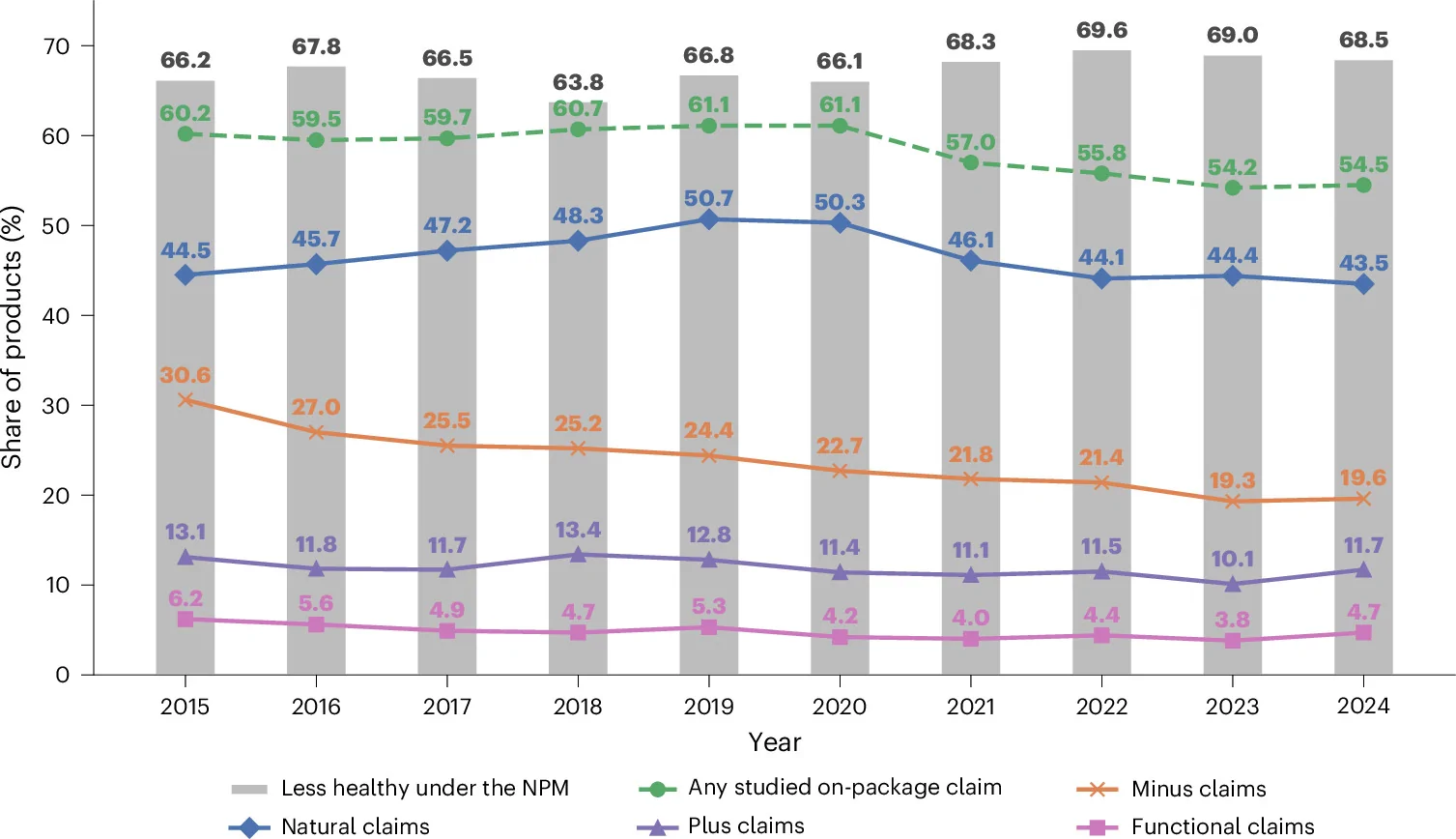
Trends in on-package claims and product nutritional quality. Yearly trends in nutritional quality and the prevalence of the four on-package claim groups from 2015 to 2024. Grey bars show the annual percentage of products classified as less healthy under the NPM (NP score ≥4 for foods; NP score ≥1 for beverages). Coloured lines show the annual percentage of products carrying at least one studied claim overall or at least one claim in each of the Minus, Plus, Natural and Functional groups.Source data are provided in Source Data Fig. 1. Source data

Figure 1 presents yearly trends in nutritional quality and the prevalence of the four types of on-package claim from 2015 to 2024. These claims remained pervasive throughout the decade, with over half of all new products carrying at least one claim, peaking at 61.1% in 2019 and 2020 before declining slightly to 54.5% in 2024. Natural claims were the most prevalent, followed by Minus and Plus claims, while Functional claims remained comparatively rare. Minus claims declined from 30.6% in 2015 to 19.6% in 2024.

Despite this widespread use of on-package claims, about two-thirds of products were classified as less healthy under the NPM. At the market level, the high prevalence of claims therefore coexisted with a high prevalence of products with less favourable overall nutritional quality.

### Prevalence of on-package claims by product category

**Fig. 2:**
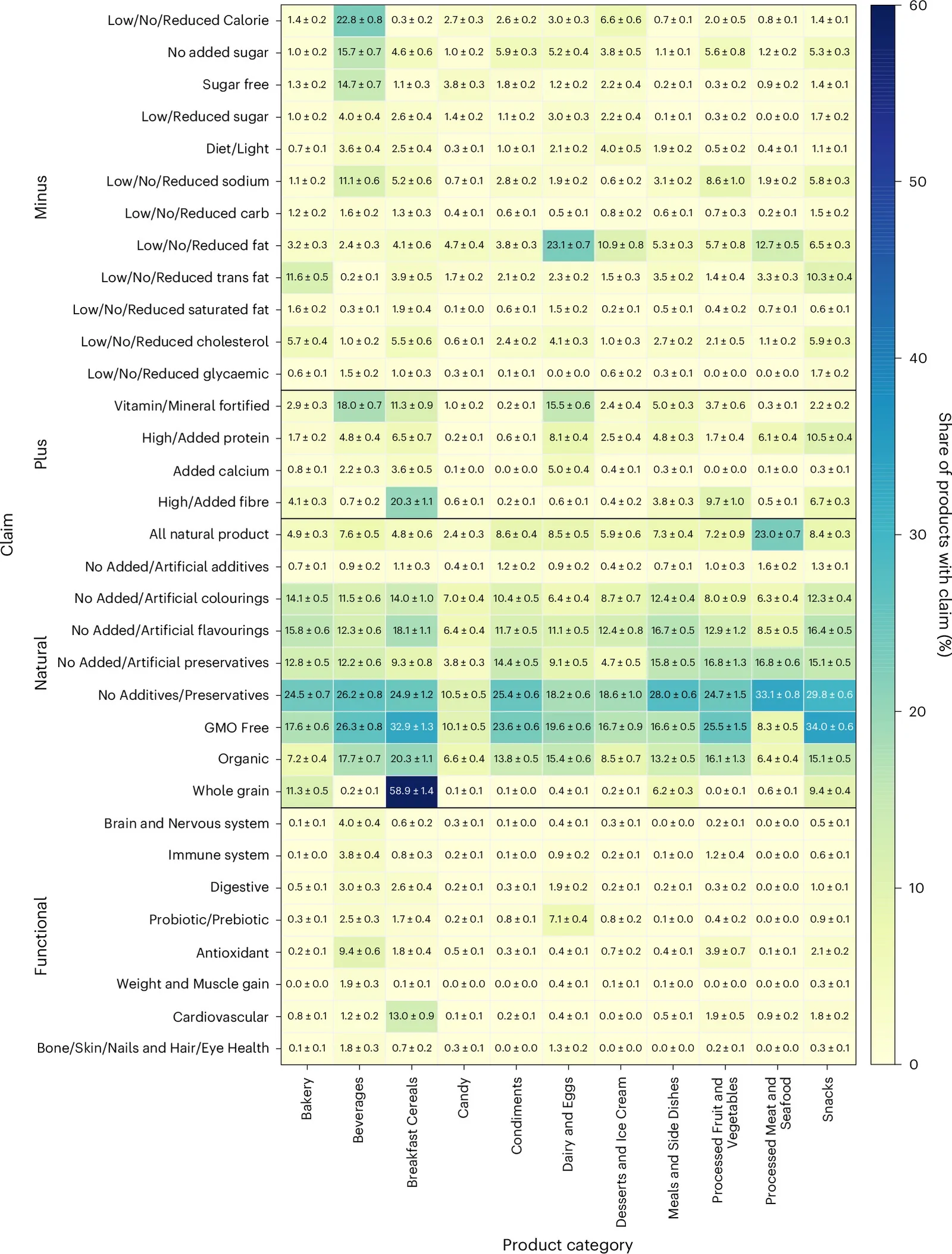
Prevalence of on-package claims by product category. The heatmap shows the prevalence of specific claims, organized into Minus, Plus, Natural and Functional claim groups, across product categories. The colour intensity, from light yellow (lower prevalence) to dark blue (higher prevalence), represents the percentage of products carrying each claim. Each cell reports the estimated percentage and its normal-approximation 95% confidence-interval as estimate ± margin. Horizontal black lines separate the four claim groups. Source data are provided in Source Data Fig. 2. Source data

The heatmap in Fig. 2 shows claim prevalence by category. Claim prevalence varies systematically across categories, with Breakfast Cereals, Beverages and Snacks exhibiting higher prevalence, and Candy, Desserts and Ice Cream, and Bakery showing lower levels. Minus claims (for example, ‘Low/No/Reduced Calorie’ and ‘No Added Sugar’) are concentrated in Beverages, Plus claims (for example, ‘High/Added Fibre’ and ‘Vitamin/Mineral Fortified’) are concentrated in Breakfast Cereals and Beverages, Natural claims (for example, ‘Whole Grain’, ‘GMO Free’ (GMO, genetically modified organism) and ‘Organic’) are the most common across categories and Functional claims (for example, ‘Cardiovascular Health’ and ‘Antioxidants’) remain rare but are concentrated in Breakfast Cereals and Beverages.

Among products with claims, the average is 2.8 claims per product. Natural claims are most frequent, while Plus claims are less common. Breakfast Cereals (3.3), Beverages (3.1), Bakery (3.0) and Snacks (3.0) show the highest density, while Candy (2.3), Desserts and Ice Cream (2.5), and Condiments (2.5) have lower claim density. These patterns indicate substantial variation in both claim prevalence and density across categories. Supplementary Fig. 1 provides additional details on claim prevalence and density by claim type and category.

### Discrepancy of on-package claims and nutritional quality of products

**Fig. 3:**
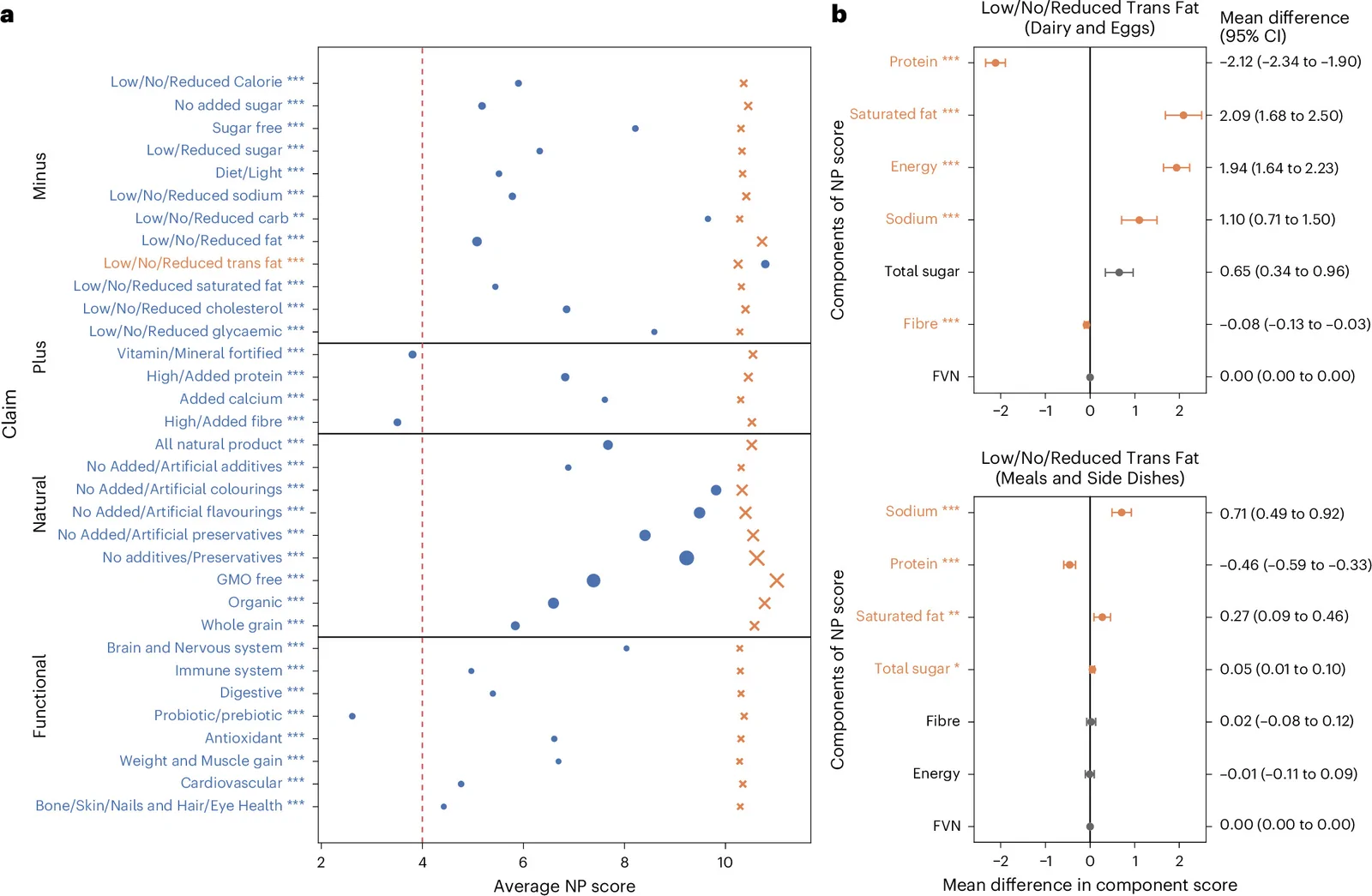
On-package claims and NP scores of food products. **a**, NP scores by claim status at the market level, comparing products with and without each claim. Blue dots and orange crosses show mean NP scores among products with and without each claim, respectively. Marker size represents claim prevalence, defined as the percentage of all food products carrying each claim. Orange claim labels with asterisks indicate discrepancy, that is, products with the claim have significantly higher, less favourable NP scores. Blue labels with asterisks indicate alignment, that is, products with the claim have significantly lower, more favourable NP scores. The red dashed line marks the NPM less healthy threshold for foods (NP score ≥4). **b**, Nutrient contributions to discrepancy within categories. The forest plots show the mean differences in NPM component scores (defined as with claim minus without claim); dots and whiskers show the mean differences and 95% Wald confidence intervals, respectively, with numerical estimates reported on the right. Orange indicates a statistically significant contribution towards a less favourable NP score (a higher A-point component or lower C-point component), and grey indicates a difference that is not statistically significant. Components are ordered by the absolute magnitude of their signed contribution. For Low/No/Reduced Trans Fat, the sample sizes with and without the claim are *n* = 339 and *n* = 14,200 in Dairy and Eggs and *n* = 749 and *n* = 20,469 in Meals and Side Dishes, respectively. FVN, fruit, vegetables and nuts. In **a** and **b**, the unit of analysis is a unique newly launched product. Asterisks indicate unadjusted two-sided Wilcoxon rank-sum (Mann–Whitney *U*) tests (*P < 0.05, **P < 0.01, ***P < 0.001). Exact *P* values, *U* statistics and group-specific sample sizes are provided in the Source Data Fig. 3. Source data

**Fig. 4:**
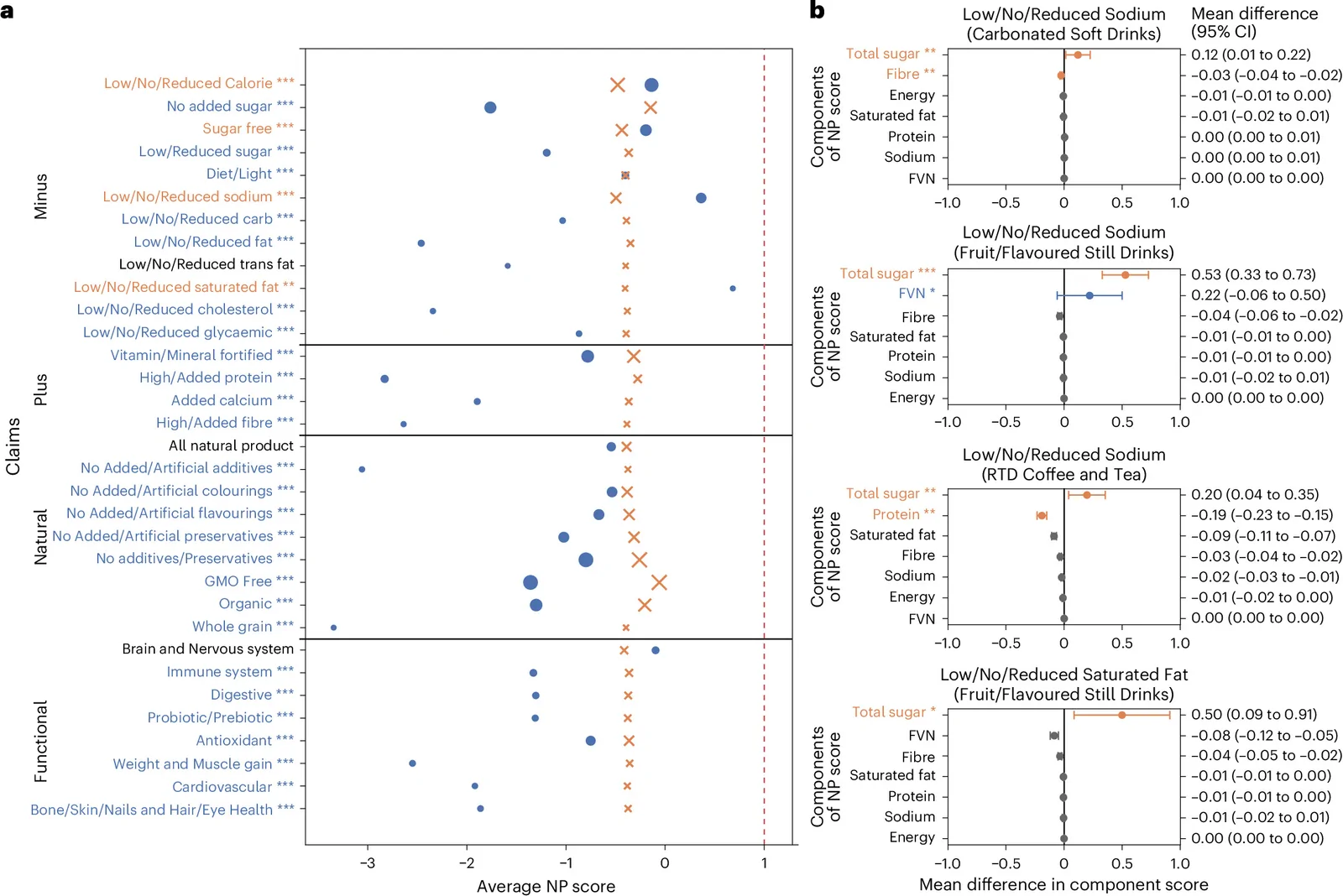
On-package claims and NP scores of beverage products. **a**, NP scores by claim status at the market level, comparing products with and without each claim. Blue dots and orange crosses show mean NP scores among products with and without each claim, respectively. Marker size represents claim prevalence, defined as the percentage of all beverage products carrying each claim. Orange claim labels with asterisks indicate discrepancy, that is, products with the claim have significantly higher, less favourable NP scores. Blue labels with asterisks indicate alignment, that is, products with the claim have significantly lower, more favourable NP scores. Black labels without asterisks indicate no statistically significant difference. The red dashed line marks the NPM less healthy threshold for beverages (NP score ≥1). **b**, Nutrient contributions to discrepancy within categories. The forest plots show the mean differences in NPM component scores (denoted as with claim minus without claim); dots and whiskers show the mean differences and 95% Wald confidence intervals, respectively, with numerical estimates reported on the right. Orange indicates a statistically significant contribution towards a less favourable NP score (a higher A-point component or lower C-point component), blue indicates a statistically significant contribution towards a more favourable score and grey indicates a difference that is not statistically significant. Components are ordered by the absolute magnitude of their signed contribution. For Low/No/Reduced Sodium, the sample sizes with and without the claim are *n* = 439 and *n* = 1,802 in Carbonated Soft Drinks, *n* = 69 and *n* = 1,082 in Fruit/Flavoured Still Drinks, and *n* = 56 and *n* = 1,627 in Ready-to-Drink (RTD) Coffee and Tea, respectively. For Low/No/Reduced Saturated Fat in Fruit/Flavoured Still Drinks, *n* = 13 products have the claim and *n* = 1,138 do not. In **a** and **b**, the unit of analysis is a unique newly launched product. Asterisks indicate unadjusted two-sided Wilcoxon rank-sum (Mann–Whitney *U*) tests (*P < 0.05, **P < 0.01, ***P < 0.001). Exact *P* values, *U* statistics and group-specific sample sizes are provided in Source Data Fig. 4. Source data

The alignment between claims and nutritional quality for food and beverages is examined in Figs. 3 and 4. A discrepancy is defined as a statistically significant (at the 5% level or better) difference in which products carrying a given claim have higher NP scores than those without the claim, whereas alignment is defined as a statistically significant difference in which products carrying the claim have lower NP scores than those without the claim. Differences that are not statistically significant are classified as having no significant difference.

Figures 3a and 4a present market-level comparisons between products with and without each claim, reflecting the overall product environment faced by consumers. For food, most claims are aligned, except for ‘Low/No/Reduced Trans Fat’. For beverages, patterns are more heterogeneous, with four claims exhibiting discrepancy: Low/No/Reduced Calorie, ‘Sugar Free’, ‘Low/No/Reduced Sodium’ and ‘Low/No/Reduced Saturated Fat’.

To disentangle these patterns, we conducted within-category analyses (Supplementary Fig. 2 for food and Supplementary Fig. 3 for beverages) and classified claims into four groups based on discrepancy status at both the market-wide and within-category levels: (1) market-wide and within-category discrepancy, (2) market-wide discrepancy only, (3) within-category discrepancy only and (4) no discrepancy. These classifications are summarized in Extended Data Tables 1 and 2.

Figures 3b and 4b present component analyses for claims exhibiting both market-wide and within-category discrepancy, focusing on the categories in which these discrepancies arise. Figure 3b focuses on Dairy and Eggs and Meals and Side Dishes for Low/No/Reduced Trans Fat. In Dairy and Eggs, higher NP scores reflect lower protein and higher saturated fat, energy and sodium, while in Meals and Side Dishes, discrepancy reflects higher sodium and saturated fat. Figure 4b shows that Low/No/Reduced Sodium discrepancy arises in Carbonated Soft Drinks, Fruit/Flavoured Still Drinks, and Ready-to-Drink (RTD) Coffee and Tea, driven mainly by higher total sugar. Low/No/Reduced Saturated Fat discrepancy arises in Fruit/Flavoured Still Drinks, where higher sugar offsets reductions in saturated fat. Overall, reductions in targeted nutrients are often offset by less favourable changes in other components.

However, alignment does not imply that products avoid classification as less healthy under the NPM. Many products with aligned claims still exceed the NPM thresholds, as indicated by the vertical cut-off lines in figures 3b and 4b, suggesting that relative improvements do not necessarily move products below the less healthy cut-off. This is particularly evident for most aligned claims in food categories, with limited exceptions for certain nutrient- and function-specific claims (for example, Vitamin/Mineral Fortified, High/Added Fibre and ‘Probiotic/Prebiotic’).

Discrepancy is observed across multiple categories, including Meals and Side Dishes, Processed Fruit and Vegetables, and Condiments, with little to no evidence in Bakery, Desserts and Ice Cream, and Candy. Natural claims exhibit consistent within-category discrepancy in both food and beverages. In food products, claims such as ‘No Added/Artificial Flavourings’, ‘No Additives/Preservatives’ and ‘No Added/Artificial Colourings’ show discrepancy in Condiments, Meals and Side Dishes, Processed Fruit and Vegetables, and Processed Meat and Seafood (Extended Data Table 1). In beverages, similar patterns are observed for No Added/Artificial Flavourings and No Additives/Preservatives in Juice and RTD Coffee and Tea, as well as for Organic in Flavoured Water and Nutritional Drinks (Extended Data Table 2).

## Discussion

The general finding of this study is that, although on-package claims are highly prevalent, they do not consistently correspond to products with more favourable overall nutritional profiles. At the market level, food products carrying claims generally had lower, more favourable NP scores than products without claims, except for those carrying Low/No/Reduced Trans Fat claims. Discrepancies were more frequent among beverage products and were concentrated among Minus claims, including Low/No/Reduced Calorie, Sugar Free, Low/No/Reduced Sodium and Low/No/Reduced Saturated Fat. Even when claims were associated with lower, more favourable NP scores, however, many products still exceeded the NPM thresholds for classification as less healthy.

The observed discrepancy patterns can be understood through two complementary mechanisms. First, formulation trade-offs may occur when reductions in targeted nutrients are accompanied by less favourable changes in other components. Second, selective signalling may occur when claims emphasize specific improvements while other nutritional characteristics remain unreported. For example, in beverages, Low/No/Reduced Sodium and Low/No/Reduced Saturated Fat claims are associated with higher total sugar content, while in food products, Low/No/Reduced Trans Fat claims are associated with higher saturated fat. Together, these patterns suggest that improvements in a single targeted nutrient may be offset by less favourable changes in other components.

These findings are consistent with earlier evidence showing that products with health- and nutrition-related claims are not consistently nutritionally superior to products without such claims. Evidence from European countries suggests that products with claims may have only marginally better NPs than products without claims, and that differences would be larger if NP criteria were used to regulate claim eligibility^33,34^. Evidence from Colombia further shows that products with nutrition-, health- and nature-related claims may still be high in nutrients of concern, supporting restrictions on claims for products exceeding nutrient thresholds^35^. Together, this earlier evidence suggests that attribute-specific claims do not necessarily correspond to overall nutritional quality and supports the potential value of profile-based approaches to align claim use with broader nutritional standards.

Importantly, these discrepancy patterns do not imply regulatory violations. For claims subject to regulatory requirements, compliance ensures accuracy for specific attributes but does not necessarily mean that claims provide a complete or balanced representation of overall nutritional quality. By design, these claims highlight selected characteristics while leaving other aspects of the product’s NP unaddressed. At the same time, the absence of a discrepancy does not imply that claims reliably signal overall nutritional quality. Even when claims are associated with lower NP scores, these relative improvements are often insufficient to move products out of the less healthy classification under the NPM. Thus, both discrepant and non-discrepant claims may provide only partial information, especially when consumers interpret them as broader indicators of healthfulness.

From a consumer perspective, the effects of on-package claims are not always aligned with their informational content. These claims may lead consumers to infer overall healthfulness from limited or attribute-specific information and may contribute to increased consumption when products are perceived as healthier than they are. This phenomenon can be understood through the halo effect, a cognitive bias whereby a favourable impression in one domain extends to other unrelated attributes^39–41^.

The strength and nature of these effects differ across claim types. Nutrient content claims signal improvement along a single dimension, but may be accompanied by substitutions in other components, leading consumers to overestimate overall nutritional quality. Earlier evidence shows that such claims can lead to the misinterpretation of product healthfulness and an increased willingness to purchase products that remain high in nutrients to limit^27^. Functional claims emphasize specific physiological or health-related benefits, reinforcing positive perceptions despite limited information about overall nutritional composition. More broadly, health-related claims can influence perceived healthfulness beyond their informational content^30,42^. Natural claims represent a particularly salient case: although they refer to production characteristics or ingredient origins rather than nutrient content, they may still be interpreted as indicators of healthfulness. Previous studies have shown that organic labelling can bias perceptions by enhancing perceived healthfulness, and that ‘natural’ labels can influence perceived product quality and health-related evaluations among less experienced consumers^43–45^.

Building on these mechanisms, the observed patterns have direct implications for nutrition policy. Sodium reduction has been a central focus of US dietary guidance and FDA initiatives. The *Dietary Guidelines for Americans, 2025–2030*^46^ emphasizes limiting added sugars, sodium and saturated fat while prioritizing overall dietary quality, and the FDA’s sodium reduction initiative^47^ promotes gradual reductions in sodium across the food supply. However, our findings suggest that targeting individual nutrients in isolation may not translate into meaningful improvements in overall nutritional quality. In beverages, sodium- and saturated fat-related claims are associated with higher total sugar, a particularly concerning pattern given the central role of added sugars in driving excess calorie intake and diet-related health risks. Similarly, in food products with discrepant Low/No/Reduced Trans Fat claims, such claims are associated with higher saturated fat, indicating substitution patterns that may not support consumers’ efforts to improve fat quality. These patterns highlight a gap between nutrient-specific policy targets and the multidimensional nature of nutritional quality.

Globally, approaches to improve the food information environment vary substantially. Interpretive labelling systems, such as traffic light labelling and Nutri-Score in Europe, provide summary indicators of overall nutritional quality and have been associated with improved consumer choices and product reformulation. Countries such as Chile and Mexico have implemented mandatory warning labels and broader restrictions on marketing and product promotion, including limits on which products may carry claims or be marketed to children. These approaches aim to align product-level information more closely with overall nutritional quality.

In the United States, manufacturers commonly use voluntary on-package claims that often emphasize selective attributes rather than overall composition; when used, many such claims are subject to regulatory requirements. Recent survey evidence shows that 83% of consumers read food labels before purchase and 78% prioritize health claims when reviewing labels, yet only 16% consider such claims highly trustworthy and 20% of consumers report difficulty interpreting nutritional information^48^. Consistent with our findings, these patterns highlight a gap between the salience of on-package claims and their effectiveness in conveying overall nutritional quality. Recent FDA actions, including the updated Healthy claim definition, which uses food-group criteria and limits on added sugars, sodium and saturated fat, and the proposed front-of-package ‘Nutrition Info’ labelling system, represent steps towards more profile-based approaches. Further alignment between claims and overall nutritional quality may be needed to better support consumer decision-making.

This study has several limitations that suggest directions for future research. First, the NPM provides a consistent and policy-relevant benchmark for evaluating overall nutritional quality and has been applied in regulatory contexts such as advertising restrictions and product placement in the UK. However, it is not directly applied to on-package claim regulation in the United States, although it has been used in research to evaluate nutritional quality in the US context^37^. Second, the analysis is based on newly launched products in the Mintel GNPD database and may not fully represent the broader food supply or products most commonly purchased by consumers and may not capture products introduced before this period. Third, the analysis is observational and descriptive and does not directly capture consumer responses to on-package claims. Future research could link product-level data to purchasing behaviour and examine how multiple claims jointly influence perceived healthfulness. While this study includes category-level analysis, it remains largely descriptive. Further research could examine product categories and claim types in greater depth, particularly where discrepancies are most pronounced, to inform more targeted regulatory or labelling interventions.

In summary, on-package claims provide imperfect signals of overall nutritional quality. Although they may reflect improvements in specific attributes, these improvements are often insufficient to change a product’s classification as less healthy under the NPM and may be accompanied by less favourable levels of other components. These findings highlight a fundamental limitation of attribute-based claims: they provide only partial signals that may be interpreted as broader indicators of healthfulness, but do not fully capture the multidimensional nature of nutritional quality. Strengthening the alignment between on-package information and overall nutritional quality, potentially through more comprehensive, profile-based approaches, may therefore support consumer decision-making and public health objectives.

## Methods

### Data source and sample construction

This study used product-level data from the Mintel GNPD, a widely used commercial dataset that tracks newly launched food and beverage products across global markets. The GNPD provides detailed information on product formulation, on-package labelling and claims, nutrition facts, ingredient lists and certification attributes, making it well suited to examine the alignment between marketing claims and nutritional quality.

All food and beverage products launched in the United States between January 2015 and December 2024 were extracted. To construct a comparable sample of processed consumer packaged goods, product groups outside the scope of nutrient profiling or not directly comparable were excluded, including fresh produce; baby food; bottled water; sweeteners, honey and syrups; oils; seasonings and stocks; products requiring reconstitution (for example, concentrates and mixes); alcoholic and hot beverages; and baking ingredients and mixes. The final analytical sample included 145,609 products spanning beverages and 10 food categories. Mapping between GNPD product classifications and analytical categories is provided in Supplementary Table 2.

### Nutritional quality assessment

Nutritional quality was assessed using the NPM developed by the UK Food Standards Agency in 2004–2005. It assigns points per 100 g of product for components to limit—energy (kJ), saturated fat (g), total sugar (g) and sodium (mg) (A points)—and subtracts points for components to encourage—FVN (%), fibre (g) and protein (g) (C points). The final NP score equals A points minus C points, with higher scores indicating lower nutritional quality. If a product scores ≥11 A points, protein points are excluded unless FVN points are ≥5, in which case adjusted C points excluding protein are used. Because the GNPD does not consistently report FVN content, we assigned FVN using reported percentages where available and, when not available, based on product category following Lim et al.^49^, assigning 100% or 0% FVN to subcategories based on typical composition. For food products, FVN was set to 100% for products in the Processed Fruit and Vegetables category and to 0% for all remaining food categories. For beverage products, where FVN content is more relevant, FVN was based on reported juice percentages where available. If juice content was missing, products in the Juice category were assigned 100% FVN. All other beverage products were assigned 0% FVN. Consistent with established thresholds, foods with NP scores ≥4 and beverages with NP scores ≥1 were classified as less healthy.

The NPM was implemented given its transparent construction, balanced consideration of components to limit and components to encourage, and its widespread regulatory and research use, including in marketing standards and reformulation policies in the UK and other jurisdictions^49^. Importantly, the model applies uniformly across all food and drink categories, enabling consistent and comparable assessment of nutritional quality across diverse product types.

### Claims classification

Terminology in food claims and labelling varies across studies and regulatory contexts, including terms such as nutrition claims, health claims, nutrient content claims, front-of-pack labelling and front-of-package nutrition labelling. To avoid confusion, this study used the term on-package claims to refer to the product-specific claims analysed here, while retaining the original terminology when referring to earlier studies or policy systems. Based on GNPD’s categorization, four types of on-package claim commonly used in marketing and labelling research were examined: Minus (reduced levels of nutrients to limit), Plus (added beneficial components), Natural (related to minimal processing or natural origin) and Functional (emphasizing physiological or health benefits).

Minus and Plus claims broadly correspond to nutrient content claims as defined by the FDA. Minus claims denote the reduction or absence of specific nutrients (for example, saturated fat, sodium, sugar, trans fat and calories), while Plus claims indicate the presence, addition or high levels of specific nutrients (for example, Vitamin/Mineral Fortified, High/Added Protein, Added Calcium and High/Added Fibre). These claims are nutrient-specific and provide direct signals about particular aspects of nutrient composition.

Natural claims are not formally defined as nutrient content or health claims by the FDA, but are widely used in food marketing. These claims emphasize attributes such as minimal processing, absence of artificial inputs or natural origin (for example, ‘No Artificial Additives’, Organic, GMO Free and Whole Grain). Although they do not directly reflect nutrient composition, they convey broader quality cues and are strongly associated with perceptions of healthfulness and product desirability in consumer decision-making.

Functional claims refer to or imply beneficial physiological or health-related effects following consumption. They broadly correspond to structure/function-type claims under FDA labelling frameworks, but also include more general or implicit functional cues commonly used in marketing. These claims may describe the role of specific ingredients or components in supporting physiological functions (for example, immune support, cardiovascular health, weight and muscle gain, probiotics and antioxidants). Some Functional claims may overlap with FDA-defined health claims when they describe a relationship between a food or food component and reduced risk of a disease or health-related condition. However, the Mintel GNPD does not provide a separate category corresponding directly to FDA-defined health claims; such claims are captured within broader Functional claim categories.

This classification reflects differences in regulatory definition and underlying mechanisms, distinguishing between claims that directly signal nutrient content (Minus and Plus) and those that convey broader or function-specific health signals (Natural and Functional). Claim categories follow Mintel GNPD definitions, which group claims based on marketing and product characteristics, and are mapped to US regulatory frameworks for interpretation. Full claim definitions and corresponding regulatory references are provided in Supplementary Table 1.

### Analytical approach

Initially, to assess whether on-package claims corresponded to improved nutritional quality, NP score distributions were compared between products with and without each claim using the two-sided Wilcoxon rank-sum (Mann–Whitney *U*) test. A claim was classified as aligned if products carrying the claim had significantly lower NP scores than those without (that is, were relatively healthier) and discrepant if products with the claim had significantly higher NP scores (*P* < 0.05). Market-level analyses were conducted separately for food and beverage products, followed by within-category analyses to assess whether aggregate patterns reflected category composition or within-category differences. Based on these comparisons, claims were classified into four groups: market-wide and within-category discrepancy, market-wide discrepancy only, within-category discrepancy only and no discrepancy. Second, the proportion of products exceeding NPM thresholds for classification as less healthy (NP score ≥4 for foods; NP score ≥1 for beverages) was evaluated for products carrying each claim. This threshold-based approach reflects the use of the NPM in regulatory contexts to identify less healthy products. Finally, to identify the nutritional drivers of observed differences, NP score variation was decomposed into component nutrients, distinguishing A-point components to limit and C-point components to encourage, with a focus on discrepant claims and the categories in which these discrepancies arose.

Data cleaning and variable construction were carried out in Stata/MP 18. Statistical analyses and figure generation were conducted using Python (v3.12.7) in Jupyter Notebook.

### Reporting summary

Further information on research design is available in the Nature Portfolio Reporting Summary linked to this article.

## Supplementary information

Supplementary Information Supplementary Tables 1 and 2 and Figs. 1–3.

Reporting Summary

Supplementary Data Source data and codebooks for Supplementary Figs. 1–3.

## Source data

Source Data Fig. 1 Statistical source data.

Source Data Fig. 2 Statistical source data.

Source Data Fig. 3 Statistical source data, including sample sizes and *P* values.

Source Data Fig. 4 Statistical source data, including sample sizes and *P* values.

## Data Availability

The data used in this study are from the Mintel GNPD, a proprietary database that requires a valid licence. Access information is available through Mintel. Due to licencing restrictions, the original GNPD data cannot be redistributed. A synthetic dataset that preserves the structure and key statistical properties of the cleaned analytical data, without containing real product-level observations, is available in the replication package via Zenodo at https://doi.org/10.5281/zenodo.22022624 (ref. ^50^). Source data are provided with this paper.

## Extended data

**Extended Data Table 1:** Classification of on-package claim discrepancy across food categories

Claim type	Specific claim	Discrepancy group	Categories with discrepancy
Minus	Low/No/Reduced Trans Fat	Market-wide and within-category discrepancy	Dairy & Eggs; Meals & Side Dishes
Minus	Diet/Light	Within-category discrepancy only	Breakfast Cereals; Condiments
Minus	Low/No/Reduced Carb	Within-category discrepancy only	Snacks
Minus	Low/No/Reduced Sodium	Within-category discrepancy only	Dairy & Eggs
Minus	No Added Sugar	Within-category discrepancy only	Processed Fruit & Vegetables
Minus	Sugar Free	Within-category discrepancy only	Condiments; Processed Meat & Seafood; Snacks
Plus	Added Calcium	Within-category discrepancy only	Breakfast Cereals; Condiments; Meals & Side Dishes
Plus	Vitamin/Mineral Fortified	Within-category discrepancy only	Breakfast Cereals; Processed Fruit & Vegetables
Natural	All Natural Product	Within-category discrepancy only	Processed Fruit & Vegetables
Natural	No Added/Artificial Colourings	Within-category discrepancy only	Condiments; Meals & Side Dishes; Processed Meat & Seafood
Natural	No Added/Artificial Flavourings	Within-category discrepancy only	Condiments; Meals & Side Dishes; Processed Fruit & Vegetables; Processed Meat & Seafood
Natural	No Added/Artificial Preservatives	Within-category discrepancy only	Candy
Natural	No Additives/Preservatives	Within-category discrepancy only	Meals & Side Dishes; Processed Fruit & Vegetables; Processed Meat & Seafood
Functional	Bone/Skin/Nails/Hair/Eye Health	Within-category discrepancy only	Meals & Side Dishes
	All remaining food claims	No discrepancy	

Discrepancy is defined as a statistically significant difference in NP Scores between products with and without a given claim, based on unadjusted two-sided Wilcoxon rank-sum (Mann–Whitney U) tests. ‘Market-wide discrepancy’ refers to discrepancy in the pooled food sample, representing the overall product environment faced by consumers. ‘Within-category discrepancy only’ indicates discrepancy in at least one separately analyzed food category, but not in the pooled food sample. ‘Market-wide and within-category discrepancy’ indicates both patterns are present. ‘No discrepancy’ indicates no discrepancy at either level.

**Extended Data Table 2:** Classification of on-package claim discrepancy across beverage categories

Claim type	Specific claim	Discrepancy group	Categories with discrepancy
Minus	Low/No/Reduced Saturated Fat	Market-wide and within-category discrepancy	Fruit/Flavoured Still Drinks
Minus	Low/No/Reduced Sodium	Market-wide and within-category discrepancy	Carbonated Soft Drinks; Fruit/Flavoured Still Drinks; RTD Coffee & Tea
Minus	Low/No/Reduced Calorie	Market-Wide discrepancy only	None
Minus	Sugar Free	Market-Wide discrepancy only	None
Minus	Low/No/Reduced Trans Fat	Within-category discrepancy only	Nutritional Drinks
Minus	No Added Sugar	Within-category discrepancy only	Juice
Plus	Added Calcium	Within-category discrepancy only	Juice; RTD Coffee & Tea
Plus	Vitamin/Mineral Fortified	Within-category discrepancy only	Juice
Natural	No Added/Artificial Colourings	Within-category discrepancy only	Juice; RTD Coffee & Tea
Natural	No Added/Artificial Flavourings	Within-category discrepancy only	Juice; RTD Coffee & Tea
Natural	No Additives/Preservatives	Within-category discrepancy only	Juice; RTD Coffee & Tea
Natural	Organic	Within-category discrepancy only	Flavoured Water; Nutritional Drinks
Functional	Bone/Skin/Nails/Hair/Eye Health	Within-category discrepancy only	Nutritional Drinks
Functional	Brain & Nervous System	Within-category discrepancy only	Nutritional Drinks
Functional	Immune System	Within-category discrepancy only	Flavoured Water
Functional	Probiotic/Prebiotic	Within-category discrepancy only	Nutritional Drinks
	All remaining beverage claims	No discrepancy	

Discrepancy is defined as a statistically significant difference in NP Scores between products with and without a given claim, based on unadjusted two-sided Wilcoxon rank-sum (Mann–Whitney U) tests. ‘Market-wide discrepancy’ refers to discrepancy in the pooled beverage sample, representing the overall beverage product environment faced by consumers. ‘Within-category discrepancy only’ indicates discrepancy in at least one separately analyzed beverage category, but not in the pooled beverage sample. ‘Market-wide and within-category discrepancy’ indicates both patterns are present. ‘No discrepancy’ indicates no discrepancy at either level.

## Code availability

The replication package, including analysis code, documentation and the synthetic dataset, is publicly available via Zenodo at https://doi.org/10.5281/zenodo.22022624 (ref. ^50^). The code includes scripts for data cleaning and construction, statistical analysis and figure generation.
