# Supplementary material for: On-package claims are imperfect signals of overall nutritional quality
Source: Nat Food. 2026 Sep 24;7(10):1011–20. doi: 10.1038/s43016-026-01435-9 (PMC13694235; doi:10.1038/s43016-026-01435-9)
Supplement: Supplementary file 1 [file 43016_2026_1435_MOESM1_ESM.pdf]

nature food

Supplementary information https://doi.org/10.1038/s43016-026-01435-9

On-package claims are imperfect signals of overall nutritional quality

In the format provided by the authors and unedited

Table of Contents

Supplementary Table S1 | Classification of On-Package Claims and Mapping to U.S. Regulatory Frameworks……………………………………………………………………………….…2

Supplementary Table S2 | Mapping Mintel GNPD Product Categories to Analytical Categories in This Study……………………………………………………………………………….....5

Supplementary Figure S1 | Claim Prevalence and Claim Density by Product Category and Claim Type…………………………………………………………………………………………8

Supplementary Figure S2 | Differences in Nutrient Profile Scores by Claim Status Within Food Categories………………………………………………………………………………………9

Supplementary Figure S3 | Differences in Nutrient Profile Scores by Claim Status Within Beverage Categories……………………………………………………………………………………...11

Supplementary Table S1 | Classification of On-Package Claims and Mapping to U.S. Regulatory Frameworks

Claim Group	Specific Claim	Definition (adapted from Mintel GNPD)	Regulatory Status (U.S.)
Minus (Nutrient Reduction / Absence)	Low/No/Reduced Calorie	Product contains reduced calories compared to a reference or is labeled low-calorie.	FDA-defined nutrient content claim (21 CFR §101.60(b))
	No Added Sugar	No sugars added during processing; naturally occurring sugars may remain.	FDA-defined nutrient content claim (21 CFR §101.60(c)(2))
	Sugar Free	Contains negligible sugar (<0.5 g per 100 g or 100 mL).	FDA-defined nutrient content claim (21 CFR §101.60(c)(1))
	Low/Reduced Sugar	Reduced or low content of total sugars compared with a reference product.	"Reduced/less sugar" is an FDA-defined relative nutrient content claim (21 CFR §101.60(c)(5)); "low sugar" is not an FDA-defined nutrient content claim term
	Diet/Light	Reduced energy content, typically through lower fat or sugar formulation.	"Light/lite" is an FDA-defined nutrient content claim (21 CFR §101.56); use of "diet" is context-dependent and not uniformly defined
	Low/No/Reduced Sodium	Sodium or salt content is reduced, or product is low-sodium/sodium-free.	FDA-defined nutrient content claim (21 CFR §101.61)
	Low/No/Reduced Carb	Formulated with fewer carbohydrates or low in carbs.	No FDA-defined nutrient content claim; not included among claims defined in 21 CFR Part 101 Subpart D
	Low/No/Reduced Fat	Fat content reduced or low compared with a standard product.	FDA-defined nutrient content claim (21 CFR §101.62(b))
	Low/No/Reduced Trans Fat	Formulated without trans fats or reduced trans-fat levels.	No standalone FDA-defined nutrient content claim category for trans-fat; trans-fat is regulated within broader fat labeling under 21 CFR §101.13 and claim provisions under 68 FR 41434 (July 11, 2003)
	Low/No/Reduced Saturated Fat	Reduced or low saturated fat content.	FDA-defined nutrient content claim (21 CFR §101.62(c))
	Low/No/Reduced Cholesterol	Cholesterol content reduced or low relative to similar products.	FDA-defined nutrient content claim (21 CFR §101.62(d))
	Low/No/Reduced Glycemic	Product has a low or reduced glycemic index/load.	No FDA-defined nutrient content claim; not included among claims defined in 21 CFR Part 101 Subpart D

Claim Group	Specific Claim	Definition (adapted from Mintel GNPD)	Regulatory Status (U.S.)
Plus (Nutrient Addition / Enhancement)	Vitamin/Mineral Fortified	Product enriched or fortified with vitamins and/or minerals beyond natural levels.	FDA-defined nutrient content claim (“fortified,” “enriched,” “added”) (21 CFR §101.54(e))
	High/Added Protein	Product formulated with added protein or highlights naturally high protein content.	“High protein” is an FDA-defined nutrient content claim (21 CFR §101.54(b)); “added protein” falls under relative nutrient content claim provisions (21 CFR §101.54(e))
	Added Calcium	Product fortified or enriched with calcium.	FDA-defined nutrient content claim (21 CFR §101.54(e))
	High/Added Fiber	Product contains high or added dietary fiber content.	“High fiber” is an FDA-defined nutrient content claim (21 CFR §101.54(b)); “added fiber” falls under relative nutrient content claim provisions (21 CFR §101.54(e))
Natural (Natural Origin / Minimal Processing)	All Natural Product	Product claimed to be made entirely or primarily from natural ingredients.	Not an FDA-defined claim category; FDA policy-based claim; subject to general labeling requirements
	No Added/Artificial Additives	No artificial or chemical additives included in formulation.	Not an FDA-defined claim category; subject to general labeling requirements; factual/voluntary labeling statement
	No Added/Artificial Colourings	Formulated without synthetic coloring agents.	Not an FDA-defined claim category; subject to general labeling requirements; factual/voluntary labeling statement; ingredient definitions governed under 21 CFR §101.22
	No Added/Artificial Flavourings	No artificial flavoring substances added.	Not an FDA-defined claim category; subject to general labeling requirements; factual/voluntary labeling statement; ingredient definitions governed under 21 CFR §101.22
	No Added/Artificial Preservatives	Highlights that no artificial preservatives were used.	Not an FDA-defined claim category; subject to general labeling requirements; factual/voluntary labeling statement; ingredient definitions governed under 21 CFR §101.22
	No Additives/Preservatives	Free from all additives and preservatives, both artificial and natural.	Not an FDA-defined claim category; subject to general labeling requirements; factual/voluntary labeling statement

Claim Group	Specific Claim	Definition (adapted from Mintel GNPD)	Regulatory Status (U.S.)
	GMO Free	Formulated without genetically modified ingredients.	Not an FDA-defined claim category; voluntary claim; subject to general labeling requirements; conceptually related to the USDA Bioengineered Food Disclosure Standard (7 CFR Part 66)
	Organic	Certified organic product meeting regional organic farming standards.	USDA-regulated claim (7 CFR Part 205); distinct from FDA claim categories
	Whole Grain	Made primarily from whole-grain ingredients or containing significant whole grains.	Not an FDA-defined claim category; subject to general labeling requirements; FDA guidance-based claim (based on FDA draft guidance on whole grain label statements)
Functional (Physiological / Health Benefit)	Brain & Nervous System	Claim that product supports brain or nervous-system function (e.g., omega-3, DHA).	FDA general labeling requirement (structure/function claims)
	Immune System	Product claimed to support immune defense or function.	FDA general labeling requirement (structure/function claims)
	Digestive	Promotes digestive health, often through fiber or probiotics.	FDA general labeling requirement (structure/function claims)
	Probiotic/Prebiotic	Contains live microorganisms or prebiotic fibers to support gut microbiota.	FDA general labeling requirement (structure/function claims)
	Antioxidant	Contains antioxidant compounds that protect cells against oxidative stress.	May function as a structure/function claim under FDA general labeling requirements or as an FDA-defined nutrient content claim when tied to antioxidant nutrient levels (21 CFR §101.54(g))
	Weight & Muscle Gain	Product supporting muscle gain, weight management, or satiety.	FDA general labeling requirement (structure/function claims)
	Cardiovascular	Claim that product benefits heart or circulatory health.	FDA general labeling requirement (structure/function claims); some claims may include disease risk reduction language and are therefore regulated separately as health claims
	Bone/Skin/Nails & Hair/Eye Health	Claim linking nutrients to bone, skin, hair, nail, or eye health.	FDA general labeling requirement (structure/function claims)

Note: Claim categories follow Mintel GNPD definitions, which group claims based on marketing and product characteristics. These categories are mapped to U.S. FDA and USDA regulatory frameworks for interpretation but do not correspond one-to-one with formal regulatory claim categories. Claims not formally defined as FDA claim categories are subject to general labeling requirements under U.S. law, including the requirement that statements be truthful and not misleading.

Supplementary Table S2 | Mapping Mintel GNPD Product Categories to Analytical Categories in This Study

GNPD Super Category	Category in this study	GNPD Category	GNPD Sub-Category	In this study
Drink	Beverages	Alcoholic Beverages	Beer, Brandy, Cider, Dark Rum, Flavoured Alcoholic Beverages, Fortified & Other Wines, Gin, Liqueur, Other Spirits, Tequila, Vodka, Whisky, White Rum, Wine	Excluded
Drink	Beverages	Carbonated Soft Drinks	Carbonated Soft Drinks	Included
Drink	Beverages	Hot Beverages	Coffee, Malt & Other Hot Beverages, Tea	Excluded
Drink	Beverages	Juice Drinks	Fruit/Flavoured Still Drinks; Juice	Included
Drink	Beverages	Juice Drinks	Nectars	Excluded
Drink	Beverages	Nutritional Drinks & Other Beverages	Nutritional & Meal Replacement Drinks	Included
Drink	Beverages	Nutritional Drinks & Other Beverages	Beverage Concentrates, Beverage Mixes, Kombucha & Other Fermented Drinks	Excluded
Drink	Beverages	RTDs	RTD (Iced) Coffee, RTD (Iced) Tea	Included
Drink	Beverages	Sports & Energy Drinks	Energy Drinks, Sports Drinks	Included
Drink	Beverages	Water	Flavoured Water	Included
Drink	Beverages	Water	Water	Excluded
Food	Baby Food	Baby Food	Baby Biscuits & Rusks, Baby Cereals, Baby Fruit Products, Desserts & Yogurts, Baby Savoury Meals & Dishes, Baby Snacks, Other Baby Food, Baby Formula (0-6 months), Baby Formula (6-12 months), Growing Up Milk (1-4 years), Growing Up Milk (4+ years), Baby Juices & Drinks	Excluded
Food	Bakery	Bakery	Bread & Bread Products, Cakes, Pastries & Sweet Goods, Savoury Biscuits/Crackers, Sweet Biscuits/Cookies	Included
Food	Bakery	Bakery	Baking Ingredients & Mixes	Excluded
Food	Breakfast Cereals	Breakfast Cereals	Cold Cereals, Hot Cereals	Included
Food	Candy	Chocolate Confectionery	Chocolate Countlines, Chocolate Tablets, Individually Wrapped Chocolate Pieces, Other Chocolate Confectionery, Seasonal Chocolate	Included

GNPD Super Category	Category in this study	GNPD Category	GNPD Sub-Category	In this study
Food	Candy	Sugar & Gum Confectionery	Boiled Sweets, Gum, Liquorice, Lollipops, Marshmallows, Medicated Confectionery, Mixed Assortments, Other Sugar Confectionery, Pastilles, Gums, Jellies & Chews, Standard & Power Mints, Sticks, Liquids & Sprays, Toffees, Caramels & Nougat	Included
Food	Condiments	Sauces & Seasonings	Cooking Sauces, Dressings & Vinegar, Mayonnaise, Other Sauces & Seasonings, Pasta Sauces, Pickled Condiments, Table Sauces	Included
Food	Condiments	Sauces & Seasonings	Oils, Seasonings, Stocks	Excluded
Food	Condiments	Savoury Spreads	Dips, Meat Pastes & Pates, Sandwich Fillers/Spreads, Savoury Vegetable Pastes/Spreads	Included
Food	Condiments	Savoury Spreads	Yeast Extracts	Excluded
Food	Condiments	Sweet Spreads	Caramel & Cream Spreads, Chocolate Spreads, Confiture & Fruit Spreads, Nut, Seed & Grain Based Spreads	Included
Food	Condiments	Sweet Spreads	Honey, Syrups	Excluded
Food	Sweeteners & Sugar	Sweeteners & Sugar	Artificial Sweeteners, Other Natural Sweeteners, Sucrose	Excluded
Food	Dairy & Eggs	Dairy	Butter, Cream, Creamers, Curd & Quark, Drinking Yogurt & Liquid Cultured Milk, Evaporated Milk, Flavoured Milk, Fresh Cheese & Cream Cheese, Hard Cheese & Semi-Hard Cheese, Liquid Dairy Other, Margarine & Other Blends, Plant Based Drinks (Dairy Alternatives), Plant Based Spoonable Yogurts (Dairy Alternatives), Processed Cheese, Shortening & Lard, Soft Cheese & Semi-Soft Cheese, Soft Cheese Desserts, Spoonable Yogurt, Sweetened Condensed Milk, White Milk	Included
Food	Dairy & Eggs	Processed Fish, Meat & Egg Products	Eggs & Egg Products	Included

GNPD Super Category	Category in this study	GNPD Category	GNPD Sub-Category	In this study
Food	Desserts & Ice Cream	Desserts & Ice Cream	Chilled Desserts, Dairy Based Ice Cream & Frozen Yogurt, Dessert Toppings, Frozen Desserts, Plant Based Ice Cream & Frozen Yogurt (Dairy Alternatives), Shelf-Stable Desserts, Water Based Ice Lollies, Pops & Sorbets	Included
Food	Meals & Side Dishes	Meals & Meal Centers	Hors d'oeuvres/Canapes, Instant Noodles, Instant Pasta, Instant Rice, Meal Kits, Pastry Dishes, Pizzas, Prepared Meals, Salads, Sandwiches/Wraps	Included
Food	Meals & Side Dishes	Side Dishes	Noodles, Pasta, Potato Products, Rice, Stuffing, Polenta & Other Side Dishes	Included
Food	Meals & Side Dishes	Soup	Wet Soup	Included
Food	Meals & Side Dishes	Soup	Dry Soup	Excluded
Food	Processed Fruit & Vegetables	Fruit & Vegetables	Fruit, Vegetables (fresh fruit and vegetables are excluded)	Included
Food	Processed Meat & Seafood	Processed Fish, Meat & Egg Products	Fish Products, Meat Products, Meat Substitutes, Poultry Products	Included
Food	Snacks	Snacks	Bean-Based Snacks, Cassava & Other Root-Based Snacks, Corn-Based Snacks, Fruit Snacks, Meat Snacks, Nuts, Other Snacks, Popcorn, Potato Snacks, Rice Snacks, Snack Mixes, Snack/Cereal/Energy Bars, Vegetable Snacks, Wheat & Other Grain-Based Snacks	Included

Note: For the within-category beverage analyses, RTD (Iced) Coffee and RTD (Iced) Tea were combined as "RTD Coffee & Tea"; Energy Drinks and Sports Drinks were combined as "Sports & Energy Drinks"; and Nutritional & Meal Replacement Drinks were labelled "Nutritional Drinks."

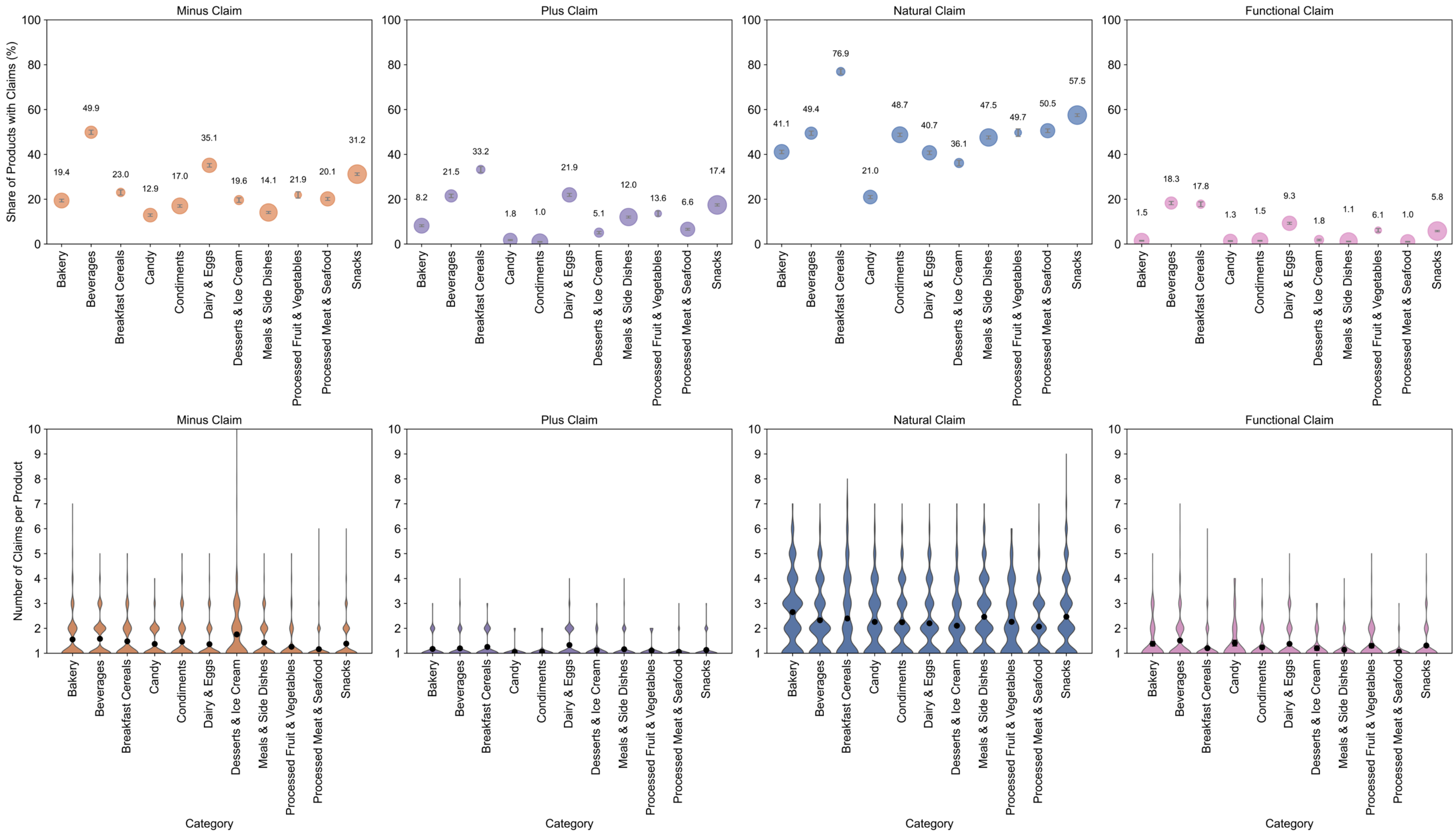

Supplementary Figure S1 | Claim Prevalence and Claim Density by Product Category and Claim Type. The upper panels show the prevalence of Minus, Plus, Natural, and Functional claims across product categories. Each dot shows the percentage of products carrying at least one claim in the respective claim group; dot size represents the total number of products in the category, and error bars show 95% Wald confidence intervals. The lower panels show the distribution of the number of claims per product among products carrying at least one claim of the respective type. Violin width represents the density of observed claim counts; black dots and whiskers show means and 95% Wald confidence intervals. Exact sample sizes and plotted estimates are provided in the Figure S1 Source Data file.

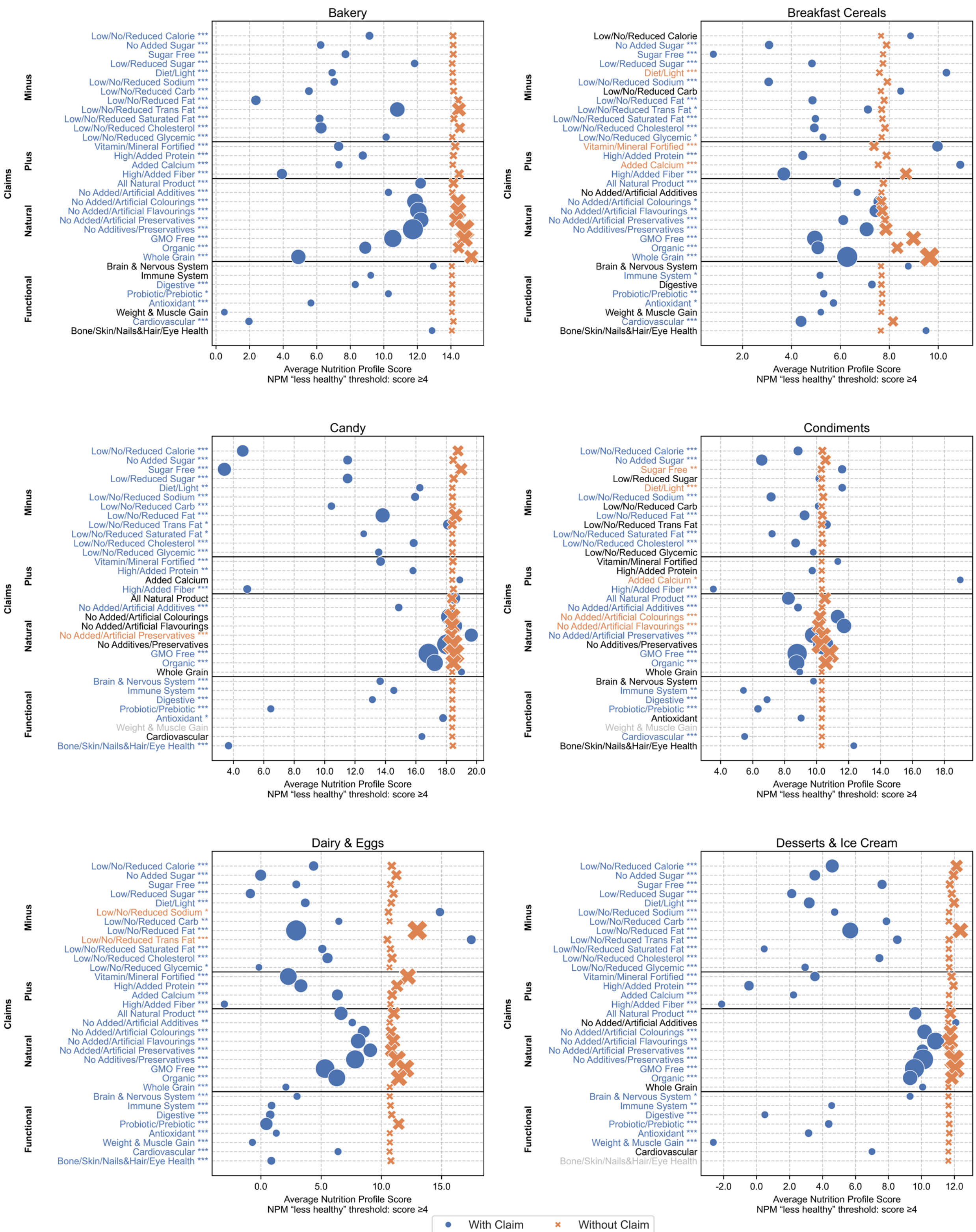

Supplementary Figure S2: Continued next page

Supplementary Figure S2: Continued

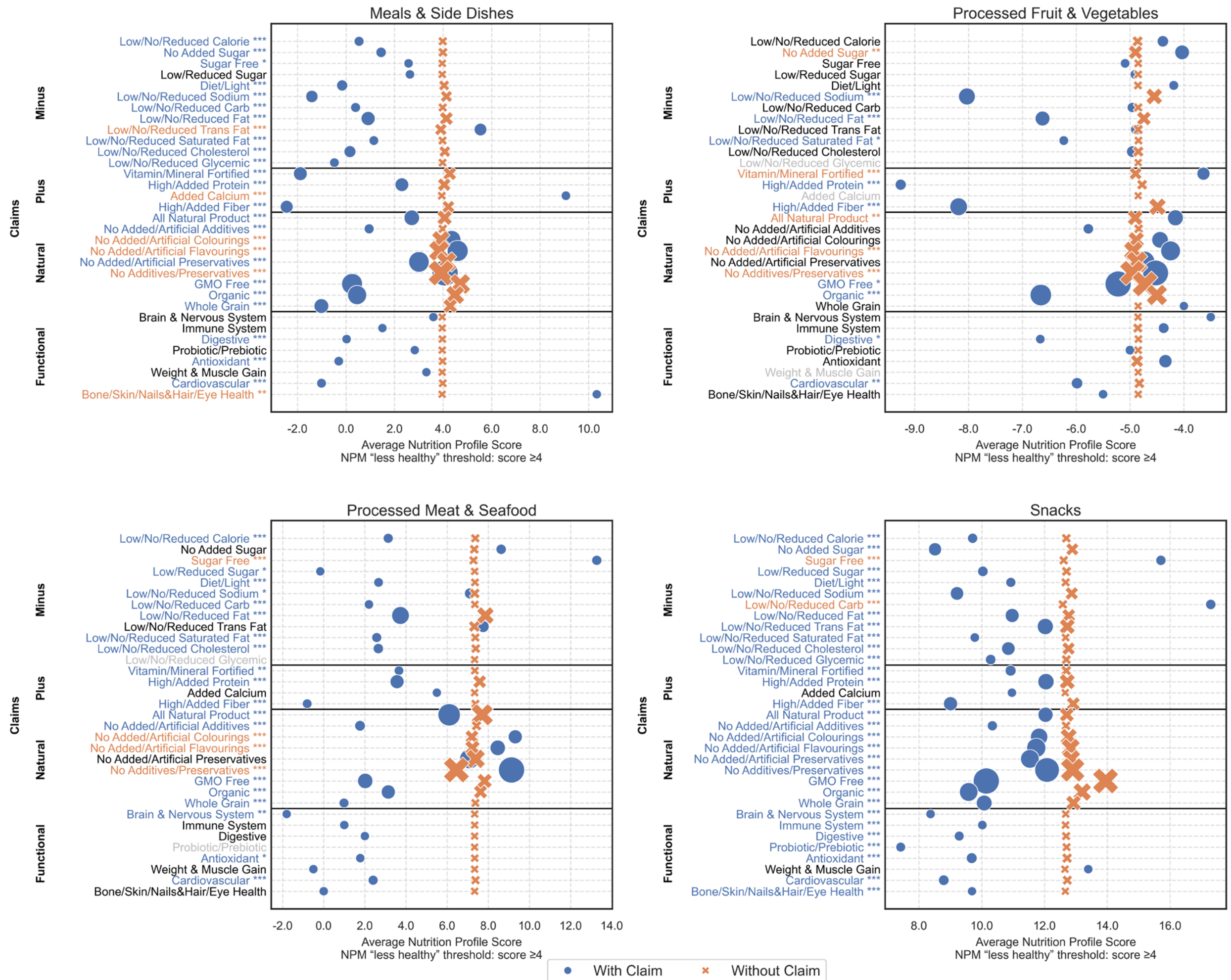

Supplementary Figure S2 | Differences in Nutrient Profile Scores by Claim Status Within Food Categories. Blue dots and orange crosses show mean Nutrient Profile (NP) Scores among products with and without each claim, respectively. Marker size represents claim prevalence, defined as the percentage of products within the respective food category carrying each claim. Orange claim labels with asterisks indicate discrepancy: products with the claim have significantly higher, less favorable NP Scores. Blue labels with asterisks indicate alignment: products with the claim have significantly lower, more favorable NP Scores. Black labels without asterisks indicate no statistically significant difference, and grey labels indicate that the comparison was not testable. The notation beneath each panel identifies the NPM "less healthy" threshold for foods (score ≥4). The unit of analysis is a unique newly launched product. Asterisks indicate unadjusted two-sided Wilcoxon rank-sum (Mann–Whitney U) tests (*P<0.05, **P<0.01, ***P<0.001). Exact P values, U statistics, and group-specific sample sizes are provided in the Figure S2 Source Data file.

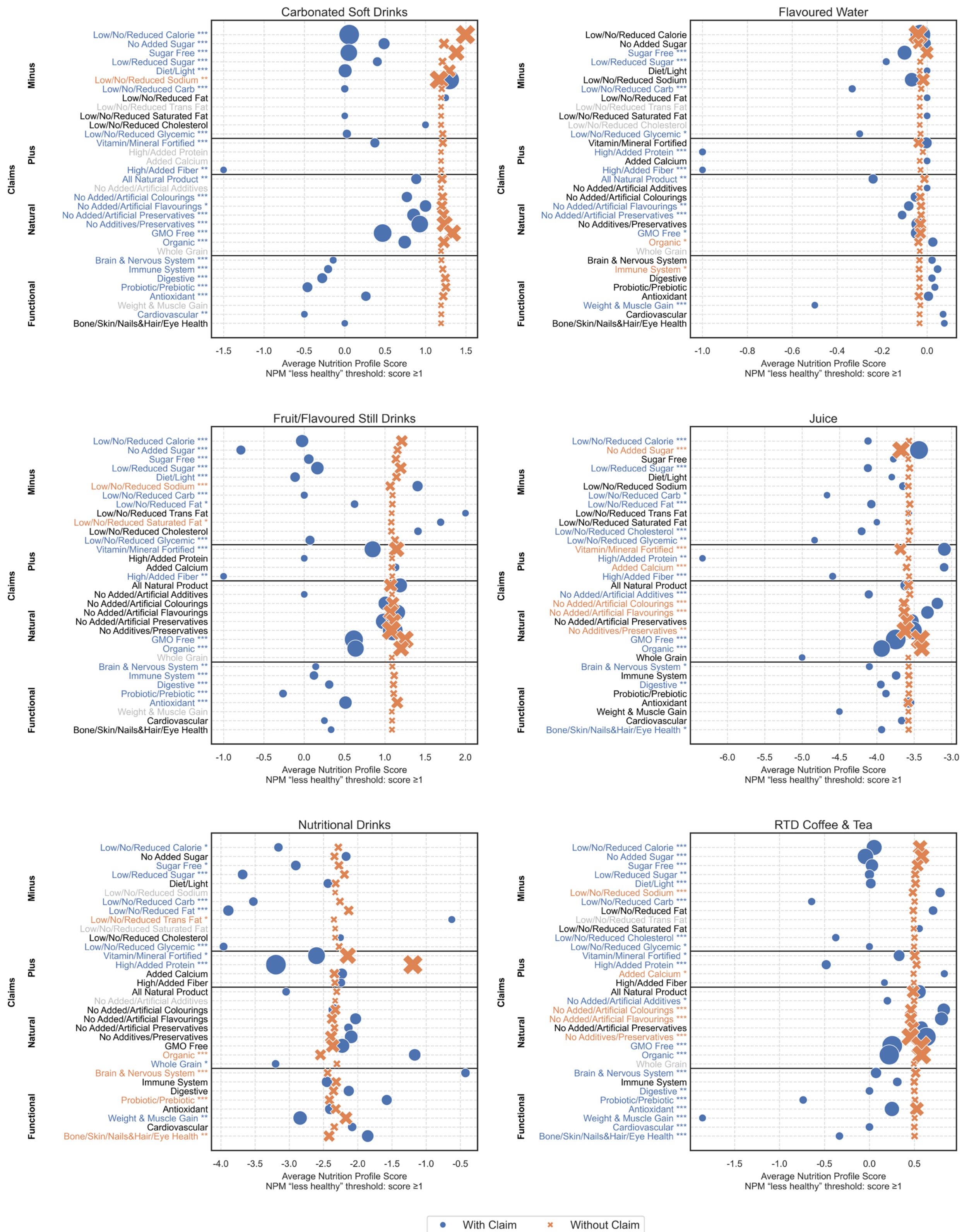

Supplementary Figure S3: Continued next page

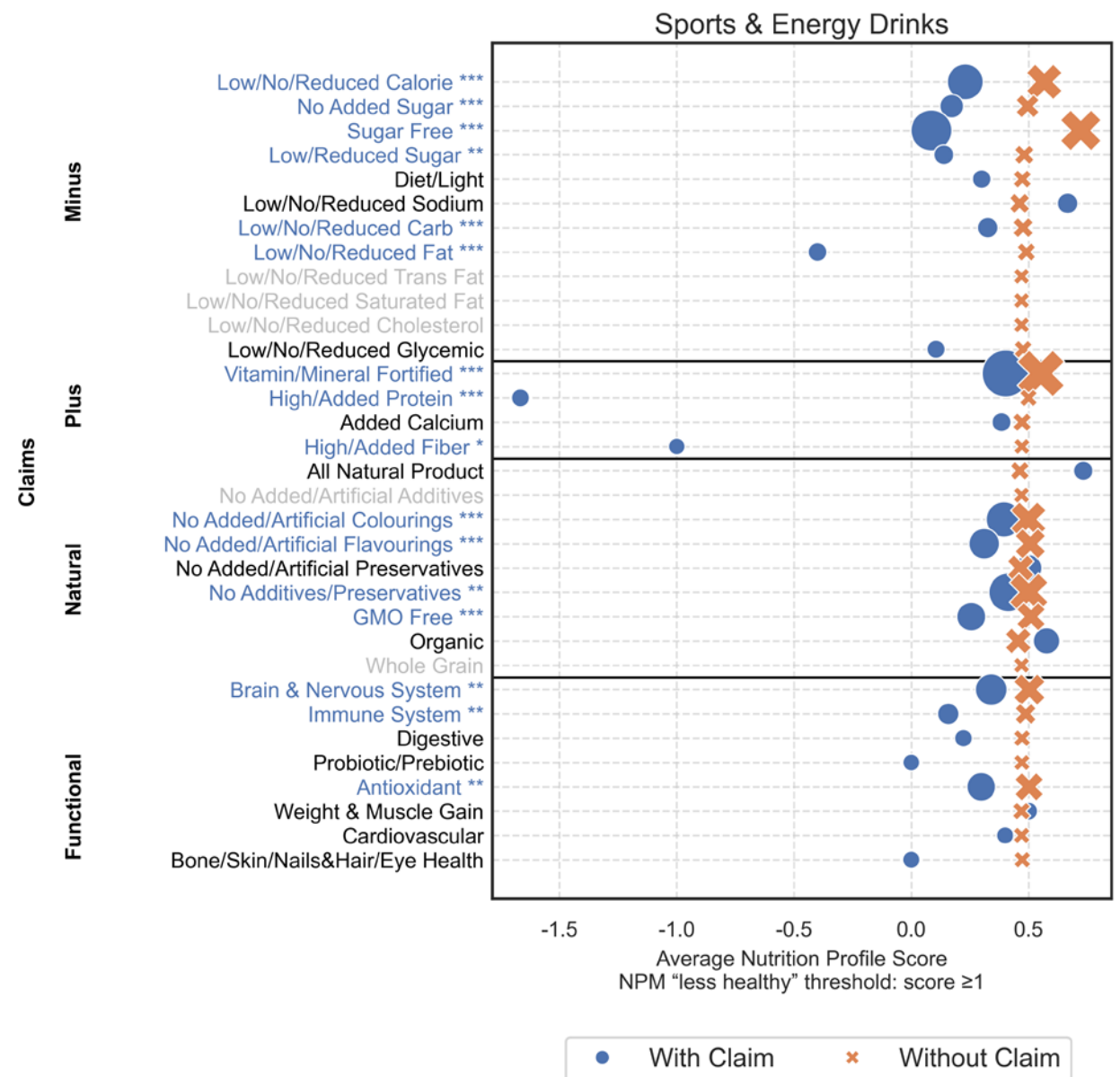

Supplementary Figure S3 | Differences in Nutrient Profile Scores by Claim Status Within Beverage Categories. Blue dots and orange crosses show mean Nutrient Profile (NP) Scores among products with and without each claim, respectively. Marker size represents claim prevalence, defined as the percentage of products within the respective beverage category carrying each claim. Orange claim labels with asterisks indicate discrepancy: products with the claim have significantly higher, less favorable NP Scores. Blue labels with asterisks indicate alignment: products with the claim have significantly lower, more favorable NP Scores. Black labels without asterisks indicate no statistically significant difference, and grey labels indicate that the comparison was not testable. The notation beneath each panel identifies the NPM "less healthy" threshold for beverages (score ≥1). The unit of analysis is a unique newly launched product. Asterisks indicate unadjusted two-sided Wilcoxon rank-sum (Mann–Whitney U) tests (*P<0.05, **P<0.01, ***P<0.001). Exact P values, U statistics, and group-specific sample sizes are provided in the Figure S3 Source Data file.
