# Supplementary material for: On-package claims are imperfect signals of overall nutritional quality
Source: Nat Food. 2026 Sep 24;7(10):1011–20. doi: 10.1038/s43016-026-01435-9 (PMC13694235; doi:10.1038/s43016-026-01435-9)
Supplement: Supplementary file 2 [file 43016_2026_1435_MOESM2_ESM.pdf]

nature portfolio

Corresponding author(s): Pei Zhou

Last updated by author(s): Aug 20, 2026

Reporting Summary

Nature Portfolio wishes to improve the reproducibility of the work that we publish. This form provides structure for consistency and transparency in reporting. For further information on Nature Portfolio policies, see our Editorial Policies and the Editorial Policy Checklist.

Statistics

For all statistical analyses, confirm that the following items are present in the figure legend, table legend, main text, or Methods section.

n/a	Confirmed	
☐	☒	The exact sample size (*n*) for each experimental group/condition, given as a discrete number and unit of measurement
☐	☒	A statement on whether measurements were taken from distinct samples or whether the same sample was measured repeatedly
☐	☒	The statistical test(s) used AND whether they are one- or two-sided<br>*Only common tests should be described solely by name; describe more complex techniques in the Methods section.*
☒	☐	A description of all covariates tested
☐	☒	A description of any assumptions or corrections, such as tests of normality and adjustment for multiple comparisons
☐	☒	A full description of the statistical parameters including central tendency (e.g. means) or other basic estimates (e.g. regression coefficient) AND variation (e.g. standard deviation) or associated estimates of uncertainty (e.g. confidence intervals)
☐	☒	For null hypothesis testing, the test statistic (e.g. *F*, *t*, *r*) with confidence intervals, effect sizes, degrees of freedom and *P* value noted<br>*Give P values as exact values whenever suitable.*
☒	☐	For Bayesian analysis, information on the choice of priors and Markov chain Monte Carlo settings
☒	☐	For hierarchical and complex designs, identification of the appropriate level for tests and full reporting of outcomes
☒	☐	Estimates of effect sizes (e.g. Cohen's *d*, Pearson's *r*), indicating how they were calculated

Our web collection on statistics for biologists contains articles on many of the points above.

Software and code

Policy information about availability of computer code

Data collection	Secondary data analysis with no software was used for data collection.
Data analysis	Data cleaning and variable construction were carried out in Stata 18 MP, and statistical analyses and figure generation were conducted using Python (v3.12.7) within Jupyter Notebook. The replication package, including analysis code, documentation, and a synthetic dataset, is available at https://doi.org/10.5281/zenodo.22022624.

For manuscripts utilizing custom algorithms or software that are central to the research but not yet described in published literature, software must be made available to editors and reviewers. We strongly encourage code deposition in a community repository (e.g. GitHub). See the Nature Portfolio guidelines for submitting code & software for further information.

Data

Policy information about availability of data

All manuscripts must include a data availability statement. This statement should provide the following information, where applicable:

- Accession codes, unique identifiers, or web links for publicly available datasets
- A description of any restrictions on data availability
- For clinical datasets or third party data, please ensure that the statement adheres to our policy

The data used in this study are sourced from the Mintel Global New Products Database (GNPD), a proprietary dataset that requires a valid license. Access information is available at: https://www.mintel.com/products/gnpd/.

Due to licensing restrictions, these data are not included in the replication package. A synthetic dataset is provided to support reproducibility of the analyses; it preserves the structure and key statistical properties of the cleaned data but does not contain real product-level observations. Replication package is available at https://doi.org/10.5281/zenodo.22022624. Source Data underlying the figures are provided with this paper.

Research involving human participants, their data, or biological material

Policy information about studies with human participants or human data. See also policy information about sex, gender (identity/presentation), and sexual orientation and race, ethnicity and racism.

Reporting on sex and gender	N/A
Reporting on race, ethnicity, or other socially relevant groupings	N/A
Population characteristics	N/A
Recruitment	N/A
Ethics oversight	N/A

Note that full information on the approval of the study protocol must also be provided in the manuscript.

Field-specific reporting

Please select the one below that is the best fit for your research. If you are not sure, read the appropriate sections before making your selection.

☐ Life sciences ☒ Behavioural & social sciences ☐ Ecological, evolutionary & environmental sciences

For a reference copy of the document with all sections, see nature.com/documents/nr-reporting-summary-flat.pdf

Behavioural & social sciences study design

All studies must disclose on these points even when the disclosure is negative.

Study description

On-package claims, including Minus, Plus, Natural, and Functional claims, are widely used to communicate nutritional or health-related benefits. This study assesses how well these claims signal overall nutritional quality. The analysis uses product-level data from the Mintel Global New Products Database (GNPD), covering packaged food and beverage products launched between 2015 and 2024. On-package claims are identified based on GNPD classifications and grouped into four categories: Minus claims, Plus claims, Functional claims, and Natural claims. Nutritional quality is assessed using the UK Nutrient Profiling Model (NPM 2004–2005).

The quantitative observational study employs a cross-sectional analytical design. Conceptually, the study examines whether on-package claims that signal improvements in specific attributes align with broader measures of overall nutritional quality. Nutrient profile scores are compared between products with and without specific claims using nonparametric Wilcoxon rank-sum (Mann–Whitney) tests due to the non-normal distribution of scores. Analyses are conducted at both the market-wide level and within product categories to improve comparability and explanatory power. In addition, a regulation and threshold-based assessment is conducted by examining the share of products exceeding NPM cutoffs for classification as "less healthy." To investigate underlying mechanisms, differences in nutrient profile scores are decomposed into their component contributions, allowing identification of patterns consistent with nutrient substitution or selective emphasis on specific attributes.
The study does not involve human participants, animal subjects, or experimental interventions.

Research sample

This study utilized product-level data from the Mintel GNPD, a widely used commercial dataset that tracks newly launched food and beverage products across global markets. GNPD provides detailed information on product formulation, on-package labeling and claims, nutrition facts, ingredient lists, and certification attributes, making it well suited for examining the alignment between marketing claims and nutritional quality.
All food and beverage products launched in the United States between January 2015 and December 2024 were extracted. To construct a comparable sample of processed consumer packaged goods, product groups outside the scope of nutrient profiling or not directly comparable were excluded, including fresh produce; baby food; bottled water; sweeteners, honey, and syrups; oils; seasonings and stocks; products requiring reconstitution (e.g., concentrates and mixes); alcoholic and hot beverages; and baking ingredients and mixes. The final analytical sample includes 145,609 products spanning beverages and 10 food categories. Mapping between GNPD product classifications and analytical categories is provided in Supplementary Table S2.

Sampling strategy

We extracted all food and drink products launched in the United States between January 2015 and December 2024 and selected the relevant products when doing the analysis, as stated in Research Sample.

Data collection

Data were downloaded as CSV files directly from the Mintel GNPD web portal following user login. Food and drink products were downloaded separately by restricting the Super-Category to "Food" or "Drink," limiting the market to the United States, and selecting products with publication dates between January 2015 and December 2024.

Timing	Data for “Drink” products were downloaded from the Mintel GNPD web portal on Apr 6, 2026, and data for “Food” products were downloaded on Apr 6, 2026.
Data exclusions	All food and beverage products launched in the United States between January 2015 and December 2024 were extracted. To construct a comparable sample of processed consumer packaged goods, product groups outside the scope of nutrient profiling or not directly comparable were excluded, including fresh produce; baby food; bottled water; sweeteners, honey, and syrups; oils; seasonings and stocks; products requiring reconstitution (e.g., concentrates and mixes); alcoholic and hot beverages; and baking ingredients and mixes. The final analytical sample includes 145,609 products spanning twelve major food and beverage categories. Mapping between GNPD product classifications and analytical categories is provided in Supplementary Table 2.
Non-participation	N/A. Participants not involved in the research.
Randomization	N/A.

Reporting for specific materials, systems and methods

We require information from authors about some types of materials, experimental systems and methods used in many studies. Here, indicate whether each material, system or method listed is relevant to your study. If you are not sure if a list item applies to your research, read the appropriate section before selecting a response.

Materials & experimental systems

n/a	Involved in the study
☒	☐ Antibodies
☒	☐ Eukaryotic cell lines
☒	☐ Palaeontology and archaeology
☒	☐ Animals and other organisms
☒	☐ Clinical data
☒	☐ Dual use research of concern
☒	☐ Plants

Methods

n/a	Involved in the study
☒	☐ ChIP-seq
☒	☐ Flow cytometry
☒	☐ MRI-based neuroimaging

Plants

Seed stocks	N/A
Novel plant genotypes	N/A
Authentication	N/A
